# Supplementary material for: Emotional Labor, Burnout, Medical Error, and Turnover Intention among South Korean Nursing Staff in a University Hospital Setting
Source: Int J Environ Res Public Health. 2021 Sep 26;18(19):10111. doi: 10.3390/ijerph181910111 (PMC8507784; doi:10.3390/ijerph181910111)
Supplement: Supplementary file 1 [file ijerph-18-10111-s001.zip › ijerph-1354558-supplementary.pdf]

Table S1. Measurement results of the study participants.

Outcomes		Measurement Results
**Emotional labor (n = 117)**	Employee-focused emotional labor	3.48 ± 0.54<br>(95% CI, 3.38 to 3.58)
	Job-focused emotional labor	3.50 ± 0.51<br>(95% CI, 3.41 to 3.60)
	**Total**	3.49 ± 0.45<br>(95% CI, 3.41 to 3.57)
**Burnout (n = 117)**	Personal burnout	3.26 ± 0.71<br>(95% CI, 3.13 to 3.39)
	Work-related burnout	3.14 ± 0.87<br>(95% CI, 2.98 to 3.30)
	Client-related burnout	2.90 ± 0.83<br>(95% CI, 2.75 to 3.05)
	**Total**	3.11 ± 0.73<br>(95% CI, 2.98 to 3.24)
**Medica error (n = 27)**	Procedure and treatment errors	n = 5, 18.5%
	Medication errors	n = 14, 51.9%
	Transfusion errors	n = 3, 11.1%
	Falls of patients	n = 11, 40.7%
	Acupuncture-related errors in KM service	n = 3, 11.1%
	Other	n = 4, 14.8%
**Turnover intention (n = 117)**	Job satisfaction domain	3.91 ± 0.78<br>(95% CI, 3.77 to 4.06)
	Job performance domain	3.79 ± 0.74<br>(95% CI, 3.65 to 3.92)
	Interpersonal relationship domain	3.69 ± 0.75<br>(95% CI, 3.56 to 3.83)
	**Total**	3.81 ± 0.65<br>(95% CI, 3.69 to 3.93)

Table S2. The results according to demographic characteristics of the study participants.

Features	Category	*n*	Emotional Labor				Burnout						Medical Error		Turnover Intention	
			**Employee-Focused Emotional Labor (Total) (M ± SD)**	***p*-Value**	**Job-Focused Emotional Labor (Total) (M ± SD)**	***p*-Value**	**PERSONAL Burnout (M ± SD)**	***p*-Value**	**Work-Related Burnout (M ± SD)**	***p*-Value**	**Client-Related Burnout (M ± SD)**	***p*-Value**	**Medical Error *N* (%) (*N* = 27)**	***p*-value**	**Turnover Intention (Total) (M ± SD)**	***p*-Value**
Age (years)	**<30**	65	3.47 ± 0.52	0.8544	3.49 ± 0.52	0.8054	3.34 ± 0.73	0.1963	3.25 ± 0.89	0.1155	2.99 ± 0.79	0.1965	18 (27.69%)	0.2695	3.87 ± 0.67	0.2342
	30≤	52	3.49 ± 0.58		3.52 ± 0.50		3.17 ± 0.68		3.00 ± 0.83		2.79 ± 0.86		9 (17.31%)		3.73 ± 0.63	
Education level	Associate degree and below	30	3.28 ± 0.38	0.0157 *	3.35 ± 0.51	0.0525	2.98 ± 0.64	0.0099 *	2.75 ± 0.71	0.0038*	2.48 ± 0.67	0.0010*	6 (20.00%)	0.8029	3.54 ± 0.62	0.0090 *
	Bachelor degree and above	87	3.55 ± 0.57		3.56 ± 0.50		3.36 ± 0.71		3.27 ± 0.88		3.04 ± 0.83		21 (24.14%)		3.90 ± 0.64	
Marriage	Unmarried	78	3.48 ± 0.55	0.8613	3.49 ± 0.50	0.6526	3.26±0.71	0.8981	3.15 ± 0.86	0.8196	2.93 ± 0.82	0.5745	18 (23.08%)	0.9999	3.89 ± 0.63	0.0729
	Married	39	3.50 ± 0.54		3.53 ± 0.54		3.27 ± 0.71		3.11 ± 0.89		2.84 ± 0.85		9 (23.08%)		3.66 ± 0.67	
Religion	No religion	72	3.48 ± 0.52	0.9752	3.44 ± 0.52	0.1177	3.31 ± 0.71	0.4079	3.23 ± 0.89	0.1774	2.97 ± 0.86	0.2396	17 (23.61%)	0.9999	3.84 ± 0.61	0.4651
	Yes	45	3.48 ± 0.59		3.60 ± 0.49		3.19 ± 0.72		3.00 ± 0.82		2.78 ± 0.76		10 (22.22%)		3.75 ± 0.72	
Monthly income	<2.5 million KRW	67	3.49 ± 0.50	0.9168	3.43 ± 0.48	0.0767	3.20 ± 0.75	0.2765	3.06 ± 0.89	0.2590	2.81 ± 0.89	0.1728	17 (25.37%)	0.5164	3.66 ± 0.67	0.0049 *
	2.5 million KRW≤	50	3.48 ± 0.60		3.60 ± 0.53		3.35 ± 0.65		3.25 ± 0.83		3.02 ± 0.72		10 (20.00%)		4.00 ± 0.59	
Clinical experiences (year)	<6	70	3.44 ± 0.50	0.2768	3.43 ± 0.50	0.0547	3.24 ± 0.73	0.6617	3.16 ± 0.91	0.7788	2.91 ± 0.86	0.8936	21 (30.00%)	0.0430 *	3.81 ± 0.69	0.9675
	6≤	47	3.55 ± 0.61		3.61 ± 0.51		3.30 ± 0.68		3.11 ± 0.81		2.89 ± 0.79		6 (12.77%)		3.81 ± 0.60	
Job position	Assistant nurse	18	3.32 ± 0.42	0.1090	3.20 ± 0.46	0.0002*	2.92 ± 0.57	0.0828	2.62 ± 0.69	0.0168*	2.30 ± 0.55	0.0026*	4 (22.22%)	0.0265 *	3.31 ± 0.50	0.0014 *
	Staff nurse	63	3.45 ± 0.52		3.45 ± 0.53		3.32 ± 0.74		3.27 ± 0.90		3.03 ± 0.84		20 (31.75%)		3.89 ± 0.67	
	Charge nurses and above	36	3.63 ±0.61		3.75 ± 0.39		3.34 ± 0.69		3.17 ± 0.82		2.96 ± 0.80		3 (8.33%)		3.91 ± 0.59	
Type of work	Shift	65	55.6	0.0650	3.50 ± 0.50	0.9794	3.18 ± 0.72	0.2876	3.03 ± 0.86	0.2240	2.78 ± 0.84	0.1608	10 (19.23%)	0.5082	3.76 ± 0.56	0.4291
	No shift	52	44.4		3.50 ± 0.52		3.33 ± 0.70		3.23 ± 0.87		2.99 ± 0.81		17 (26.15%)		3.85 ± 0.72	
Working department	WM OPD & IPD (IM, surgical, OR)	63	3.50 ± 0.51	0.8460	3.61 ± 0.49	0.0166 *	3.32 ± 0.75	0.4313	3.26 ± 0.90	0.2351	3.00 ± 0.87	0.3670	18 (28.57%)	0.2591	3.94 ± 0.62	0.0686
	ER & ICU	18	3.57 ± 0.42		3.25 ± 0.55		3.08 ± 0.59		2.94 ± 0.71		2.60 ± 0.56		1 (5.56%)		3.83 ± 0.46	
	NCISW	21	3.41 ± 0.72		3.49 ± 0.48		3.29 ± 0.73		3.15 ± 0.86		2.94±0.83		4 (19.05%)		3.57 ± 0.89	
	KM OPD & IPD	12	3.44 ± 0.56		3.23 ± 0.36		3.03 ± 0.61		2.69 ± 0.83		2.67 ± 0.87		3 (25.00%)		3.47 ± 0.34	

	Other	3	3.28 ± 0.58		3.83 ± 0.35		3.66 ± 0.43		3.28 ± 0.67		3.00 ± 0.67		1 (33.33%)		3.83 ± 0.20	
The department where they desired to work	No	39	3.63 ± 0.50	0.0350 *	3.66 ± 0.45	0.0185 *	3.46 ± 0.71	0.0289*	3.44 ± 0.80	0.0070*	3.09 ± 0.70	0.0712	10 (25.64%)	0.6481	3.95 ± 0.54	0.0907
	Yes	78	3.41 ± 0.55		3.42 ± 0.52		3.16 ± 0.69		2.99 ± 0.87		2.80 ± 0.87		17 (21.79%)		3.74 ± 0.70	
The department where they satisfy to work	No	35	3.63 ± 0.50	0.0576	3.63 ± 0.51	0.0700	3.61 ± 0.63	0.0005*	3.65 ± 0.72	<0.0001*	3.32 ± 0.79	0.0002*	9 (25.71%)	0.6408	4.08 ± 0.64	0.0030 *
	Yes	82	3.42 ± 0.55		3.45 ± 0.50		3.12 ± 0.69		2.92±0.84		2.72 ± 0.78		18 (21.95%)		3.69 ± 0.63	
Turnover experience	No	97	3.49 ± 0.55	0.8792	3.53 ± 0.51	0.3025	3.27 ± 0.74	0.7008	3.16 ± 0.91	0.6043	2.97 ± 0.82	0.0536	22 (22.68%)	0.7779	3.87 ± 0.66	0.0261 *
	Yes	20	3.47±0.50		3.40 ± 0.48		3.21 ± 0.55		3.05 ± 0.66		2.57 ± 0.78		5 (25.00%)		3.52 ± 0.55	
Department transfer experience	No	68	3.42 ± 0.48	0.1368	3.38 ± 0.53	0.0017 *	3.19 ± 0.67	0.1879	3.05 ± 0.86	0.2032	2.78 ± 0.81	0.0719	18 (26.47%)	0.3763	3.77 ± 0.69	0.4685
	Yes	49	3.57 ± 0.61		3.67 ± 0.42		3.36 ± 0.76		3.26 ± 0.88		3.06 ± 0.83		9 (18.37%)		3.86 ± 0.61	
Subjective health status	Bad and below	27	3.69 ± 0.46	0.0073 *	3.68 ± 0.41	0.0597	3.80 ± 0.57	<0.0001 *	3.75 ± 0.72	<0.0001 *	3.24 ± 0.80	0.0011 *	11(40.74%)	0.0472 *	3.92 ± 0.84	0.2172
	Normal	67	3.50±0.50		3.49 ± 0.53		3.25±0.61		3.12 ± 0.78		2.93 ± 0.79		11 (16.42%)		3.83 ± 0.59	
	Good and above	23	3.21±0.67		3.34 ± 0.50		2.66±0.64		2.47 ± 0.79		2.40 ± 0.74		5 (21.74%)		3.61 ± 0.56	

* $p < 0.05$.

Table S3. The results according to demographic characteristics of the study participants (95% CI).

Features	Category	*n*	**Emotional labor**				**Burnout**						**Turnover Intention**	
			**Employee-Focused Emotional Labor (Total) (95% CI)**		**Job-Focused Emotional Labor (Total) (95% CI)**		**Personal Burnout (95% CI)**		**Work-Related Burnout (95% CI)**		**Client-Related Burnout (95% CI)**		**Turnover Intention (Total) (95% CI)**	
Age (years)	**<30**	65	(3.35, 3.60)	−0.02 (−0.22, 0.18)	(3.36, 3.62)	−0.02 (−0.21, 0.17)	(3.16, 3.52)	0.17 (−0.09, 0.43)	(3.03, 3.47)	0.25 (−0.06, 0.57)	(2.79, 3.18)	0.20 (−0.10, 0.50)	(3.71, 4.04)	0.15 (−0.10, 0.39)
	30≤	52	(3.33, 3.65)		(3.38, 3.65)		(2.98, 3.36)		(2.77, 3.23)		(2.55, 3.03)		(3.55, 3.90)	
Education level	Associate degree and below	30	(3.13, 3.42)	−0.28 (−0.50, -0.05)	(3.16, 3.54)	−0.21 (−0.42, 0.00)	(2.74, 3.21)	−0.38 (−0.68, −0.09)	(2.48, 3.01)	−0.53 (−0.88, -0.17)	(2.23, 2.73)	−0.56 (−0.90, −0.23)	(3.31, 3.77)	−0.36 (−0.62, -0.09)
	Bachelor degree and above	87	(3.43, 3.68)		(3.45, 3.66)		(3.21, 3.51)		(3.09, 3.46)		(2.87, 3.22)		(3.76, 4.04)	
Marriage	Unmarried	78	(3.35, 3.60)	−0.02 (−0.23, 0.19)	(3.38, 3.60)	−0.05 (−0.24, 0.15)	(3.10, 3.42)	−0.02 (−0.29, 0.26)	(2.96, 3.35)	0.04 (−0.30, 0.38)	(2.74, 3.11)	0.09 (−0.23, 0.41)	(3.74, 4.03)	0.23 (−0.02, 0.48)
	Married	39	(3.32, 3.67)		(3.36, 3.71)		(3.04, 3.51)		(2.83, 3.40)		(2.56, 3.11)		(3.66, 3.87)	
Religion	No religion	45	(3.36, 3.60)	0.00 (−0.21, 0.20)	(3.32, 3.57)	−0.15 (−0.34,0.04)	(3.14, 3.47)	0.11 (−0.15, 0.38)	(3.02, 3.44)	0.22 (-0.10, 0.55)	(2.77, 3.17)	0.19 (−0.13, 0.50)	(3.70, 3.99)	0.09 (−0.16, 0.34)
	Yes	72	(3.31, 3.66)		(3.45, 3.74)		(2.98, 3.41)		(2.76, 3.25)		(2.56, 3.01)		(3.54, 3.97)	
Monthly income	<2.5 million KRW	67	(3.37, 3.61)	0.01 (−0.19, 0.21)	(3.31, 3.55)	−0.17 (−0.36, 0.02)	(3.02, 3.38)	−0.15 (−0.41, 0.12)	(2.84, 3.28)	−0.18 (−0.51, 0.14)	(2.59, 3.03)	−0.21 (−0.52, 0.09)	(3.50, 3.83)	−0.34 (−0.57, −0.11)
	2.5 million KRW≤	50	(3.31, 3.65)		(3.45, 3.75)		(3.16, 3.53)		(3.01, 3.48)		(2.82, 3.22)		(3.84, 4.17)	
Clinical experiences (year)	<6	70	(3.32, 3.56)	-0.11 (−0.31, 0.09)	(3.31, 3.55)	-0.18 (−0.37, 0.00)	(3.06, 3.41)	−0.06 (−0.33, 0.21)	(2.94, 3.38)	0.05 (−0.28, 0.37)	(2.70, 3.11)	0.02 (−0.29, 0.33)	(3.65, 3.98)	0.01 (−0.24, 0.25)
	6≤	47	(3.37, 3.73)		(3.46, 3.76)		(3.10, 3.50)		(2.87, 3.35)		(2.65, 3.12)		(3.63, 3.98)	
Job position	Assistant nurse	18	(3.11, 3.52)	−0.13 (−0.48, 0.21)	(2.97, 3.43)	−0.25 (−0.56, 0.06)	(2.64, 3.20)	−0.39 (−0.85, 0.06)	(2.27, 2.96)	-0.66 (−1.20, −0.11)	(2.02, 2.57)	−0.74 (−1.25, −0.22)	(3.06, 3.55)	−0.59 (−0.99, -0.18)
	Staff nurse	63	(3.32, 3.58)	-0.18 (-0.45, 0.10)	(3.31, 3.58)	−0.31 (−0.55, −0.07)	(3.12, 3.50)	−0.03 (−0.38, 0.33)	(3.05, 3.50)	0.11 (−0.32, 0.54)	(2.82, 3.25)	0.07 (−0.33, 0.47)	(3.73, 4.06)	−0.02 (−0.34, 0.30)
	Charge nurses and above	36	(3.42, 3.83)	−0.31 (−0.69, 0.07)	(3.62, 3.89)	−0.56 (−0.89, −0.22)	(3.11, 3.57)	-0.42 (-0.91, 0.07)	(2.89, 3.44)	−0.55 (−1.14, 0.05)	(2.69, 3.23)	−0.67 (−1.22, -0.11)	(3.72, 4.11)	−0.61 (−1.04, -0.17)
Type of work	Shift	52	(3.45, 3.72)	0.19 (−0.01, 0.38)	(3.36, 3.64)	0.00 (−0.19, 0.19)	(2.98, 3.39)	−0.14 (−0.40, 0.12)	(2.79, 3.27)	−0.20 (−0.52, 0.12)	(2.55, 3.01)	−0.22 (−0.52, 0.09)	(3.60, 3.91)	−0.10 (−0.34, 0.14)
	No shift	65	(3.26, 3.54)		(3.38, 3.63)		(3.15, 3.50)		(3.01, 3.44)		(2.79, 3.20)		(3.67, 4.03)	
Working department	WM OPD & IPD (IM, surgical,	63	(3.37, 3.63)		(3.49, 3.74)		(3.14, 3.51)		(3.04, 3.50)		(2.78, 3.23)		(3.78, 4.10)	

	OR)													
	ER & ICU	18	(3.35, 3.78)		(2.98, 3.53)		(2.79, 3.38)		(2.59, 3.30)		(2.32, 2.88)		(3.61, 4.07)	
	NCISW	21	(3.08, 3.73)		(3.27, 3.71)		(2.96, 3.63)		(2.76, 3.55)		(2.56, 3.32)		(3.16, 3.98)	
	KM OPD & IPD	12	(3.09, 3.80)		(3.00, 3.46)		(2.64, 3.43)		(2.16, 3.23)		(2.13, 3.23)		(3.25, 3.70)	
	Other	3	(1.83, 4.72)		(2.95, 4.73)		(2.59, 4.74)		(1.61, 4.95)		(1.34, 4.66)		(3.32, 4.35)	
The department where they desired to work	No	39	(3.47, 3.80)		(3.51, 3.81)		(3.23, 3.70)		(3.19, 3.70)		(2.87, 3.32)		(3.78, 4.13)	
	Yes	78	(3.28, 3.53)	0.22 (0.02, 0.43)	(3.31, 3.54)	0.23 (0.04, 0.43)	(3.01, 3.32)	0.30 (0.03, 0.57)	(2.79, 3.18)	0.46 (0.13, 0.78)	(2.60, 3.00)	0.29 (-0.03, 0.61)	(3.58, 3.89)	0.22 (−0.03, 0.47)
The department where they satisfy to work	No	35	(3.46, 3.80)		(3.46, 3.81)		(3.39, 3.82)		(3.41, 3.90)		(3.05, 3.60)		(3.86, 4.30)	
	Yes	82	(3.30, 3.54)	0.21 (−0.01, 0.42)	(3.34, 3.56)	0.19 (−0.02, 0.39)	(2.96, 3.27)	0.49 (0.22, 0.76)	(2.74, 3.11)	0.73 (0.41, 1.05)	(2.55, 2.89)	0.61 (0.29, 0.92)	(3.56, 3.83)	0.39 (0.13, 0.64)
Turnover experience	No	97	(3.37, 3.60)	0.02	(3.42, 3.63)	0.13	(3.12, 3.42)	0.07	(2.98, 3.34)	0.11	(2.80, 3.13)	0.39	(3.74, 4.00)	0.36
	Yes	20	(3.23, 3.70)	(−0.24, 0.29)	(3.17, 3.62)	(−0.12, 0.38)	(2.95, 3.46)	(−0.28, 0.41)	(2.74, 3.36)	(−0.31, 0.54)	(2.21, 2.94)	(-0.01, 0.79)	(3.26, 3.77)	(0.04, 0.67)
Department transfer experience	No	68	(3.30, 3.54)	0.15	(3.25, 3.51)	-0.30	(3.03, 3.35)	−0.18	(2.85, 3.26)	−0.21	(2.59, 2.98)	-0.28	(3.61, 3.94)	-0.09
	Yes	49	(3.40, 3.75)	(−0.35, 0.05)	(3.55, 3.80)	(−0.48, −0.11)	(3.15, 3.58)	(−0.44, 0.09)	(3.01, 3.51)	(−0.53, 0.11)	(2.82, 3.30)	(-0.58, 0.03)	(3.69, 4.04)	(-0.33, 0.15)
Subjective health status	Bad and below	27	(3.51, 3.87)	0.19 (−0.10, 0.48)	(3.51, 3.84)	0.19 (−0.09, 0.47)	(3.58, 4.03)	0.56 (0.22, 0.89)	(3.47, 4.04)	0.63 (0.20, 1.05)	(2.92, 3.56)	0.31 (-0.13, 0.74)	(3.59, 4.25)	0.09 (-0.27, 0.45)
	Normal	67	(3.37, 3.62)	0.29 (−0.02, 0.59)	(3.36, 3.62)	0.15 (−0.14, 0.44)	(3.10, 3.40)	0.58 (0.23, 0.94)	(2.93, 3.31)	0.65 (0.20, 1.10)	(2.74, 3.13)	0.53 (0.07, 1.00)	(3.69, 3.98)	0.22 (-0.16, 0.61)
	Good and above	23	(2.92, 3.50)	0.48 (0.11, 0.84)	(3.12, 3.55)	0.34 (−0.01, 0.68)	(2.39, 2.94)	1.14 (0.72, 1.56)	(2.13, 2.81)	1.28 (0.75, 1.81)	(2.08, 2.72)	0.84 (0.30, 1.38)	(3.37, 3.85)	0.31 (-0.13, 0.76)

Table S4. Influence of employee-focused emotional labor on burnout, medical error, and turnover intention ($N = 117$).

	**Burnout Parameter Estimates (*p*-Value)**				**Medical Error Parameter Estimates (*p*-value)**	**Turnover Intention Parameter Estimates (*p*-value)**
	**Personal**	**Work-Related**	**Client-Related**	**Total**		
F-value	**4.08**	4.76	3.78	5.04		2.33
p-value	<0.0001	<0.0001	<0.0001	<0.0001		0.0057
R^2	0.395	0.433	0.377	0.446		0.272
Adj R^2	0.298	0.342	0.277	0.358		0.155
Intercept (Constant)	2.060 (0.0016)	1.52 (0.0467)	0.648 (0.3902)	1.449 (0.0228*)	−5.529 (0.0484)	3.200 (<0.0001)
Clinical experience (years)						
≤6	0.092 (0.7174)	0.034 (0.911)	0.232 (0.4422)	0.116 (0.6462)	0.570 (0.5996)	−0.115 (0.6550)
>6	-	-	-	-	-	-
Education level						
Bachelor degree or above	0.192 (0.2709)	0.234 (0.2578)	0.250 (0.2261)	0.224 (0.1932)	0.154 (0.8402)	0.047 (0.7897)
Junior college or below	-	-	-	-	-	-
Monthly income (millions of KRW/month)						
≤2.5	0.063 (0.6489)	0.126 (0.443)	0.134 (0.4118)	0.105 (0.4418)	0.052 (0.9350)	0.216 (0.1222)
>2.5	-	-	-	-	-	-
Job position						
Assistant nurse	−0.059 (0.8270)	−0.038 (0.905)	−0.057 (0.8571)	−0.052 (0.8452)	1.388 (0.2723)	−0.316 (0.2460)
Staff nurse	0.100 (0.7201)	0.243 (0.4657)	0.452 (0.1741)	0.254 (0.3597)	2.352 (0.0650)	−0.058 (0.8388)
Charge nurse or above	-	-	-	-	-	-
Department						
WM OPD & IPD	−0.012 (0.9754)	0.374 (0.4009)	0.126 (0.7770)	0.154 (0.6775)	−0.226 (0.8741)	0.304 (0.4232)
ER & ICU	−0.143 (0.7178)	0.25 (0.5946)	−0.264 (0.5722)	−0.053 (0.8912)	−2.425 (0.1716)	0.216 (0.5886)
NCISW	0.010 (0.9793)	0.344 (0.4644)	0.163 (0.7285)	0.165 (0.6725)	−0.788 (0.6164)	0.028 (0.9450)
KM OPD & IPD	−0.227 (0.5777)	−0.072 (0.881)	−0.015 (0.9753)	−0.112 (0.7812)	−0.291 (0.8534)	−0.037 (0.9279)
Other	-	-	-	-	-	-
Desire to work in current department						
Yes	−0.024 (0.8535)	−0.098 (0.5302)	0.065 (0.6762)	−0.020 (0.8795)	0.296 (0.6047)	−0.054 (0.6862)
No						
Satisfaction with working in current department						
Yes	−0.275 (0.0558)	−0.469 (0.0063)	−0.298 (0.0788)	−0.346 (0.0151*)	0.098 (0.8692)	−0.238 (0.0998)
No	-	-	-	-	-	-
Turnover experience						
Yes	0.083 (0.5989)	0.152 (0.4184)	−0.173 (0.3555)	0.026 (0.8694)	0.899 (0.2255)	−0.171 (0.2865)

No	-	-	-	-	-	-
Department transfer experience						
Yes	−0.022 (0.8893)	0.045 (0.8076)	0.149 (0.4164)	0.053 (0.7303)	−0.210 (0.7579)	0.045 (0.7750)
No	-	-	-	-	-	-
Self-rated health						
Bad or below	0.898 (<0.0001)	0.974 (<0.0001)	0.411 (0.0652)	0.767 (<0.0001*)	0.581 (0.4658)	0.152 (0.4209)
Normal	0.443 (0.0051)	0.473 (0.0112)	0.354 (0.0555)	0.424 (0.0065*)	−0.411 (0.5697)	0.152 (0.3311)
Good or above	-	-	-	-	-	-
Employee-focused emotional labor	0.213 (0.0673)	0.235 (0.0889)	0.419 (0.0027*)	0.284 (0.0141*)	0.731 (0.1746)	0.144 (0.2198)

Abbreviations. AN, assistant nurse; ER, emergency room; ICU, intensive care unit; IPD, inpatient department; KM, Korean medicine; NCISW, nursing care integrated service ward; OPD, outpatient department; WM, Western medicine. * $p < 0.05$.

Table S5. Influence of job-focused emotional labor on burnout, medical error, and turnover intention (*N* = 117).

	Burnout Parameter Estimates (*p*-Value)				Medical Error Parameter Estimates (*p*-Value)	Turnover Intention Parameter Estimates (*p*-Value)
	Personal	Work-Related	Client-Related	Total		
F-value	**3.85**	4.46	2.94	2.58		2.58
p-value	<0.0001	<0.0001	0.0005	0.0022		0.0022
R^2	0.381	0.417	0.320	0.292		0.292
Adj R^2	0.282	0.323	0.212	0.179		0.179
Intercept	2.194 (0.0043)	2.024 (0.0255)	1.597 (0.0846)	1.955 (0.0108)	−7.414 (0.0227)	2.543 (0.0008 *)
Clinical experience (years)						
≤6	0.083 (0.7491)	−0.001 (0.9981)	0.167 (0.5968)	0.081 (0.7552)	0.578 (0.5970)	−0.071 (0.7806)
>6	-	-	-	-	-	-
Education level						
Bachelor degree or above	0.244 (0.1606)	0.298 (0.1500)	0.364 (0.0873)	0.299 (0.0869)	0.217 (0.7718)	0.069 (0.6862)
Junior college or below	-	-			-	-
Income (millions of KRW/month)						
≤2.5	0.021 (0.8806)	0.083 (0.6146)	0.058 (0.7318)	0.052 (0.7083)	−0.115 (0.8546)	0.181 (0.1848)
>2.5	-	-	-	-	-	-
Job position						
Assistant nurse	−0.034 (0.9032)	−0.058 (0.8609)	−0.099 (0.7720)	−0.062 (0.8251)	1.700 (0.1799)	−0.200 (0.4670)
Staff nurse	0.084 (0.7716)	0.174 (0.6116)	0.324 (0.3584)	0.186 (0.5208)	2.502 (0.0501)	0.035 (0.9009)
Charge nurse or above	-	-	-	-	-	-
Department						
WM OPD & IPD	0.081 (0.8303)	0.472 (0.2932)	0.299 (0.5157)	0.273 (0.4699)	0.073 (0.9577)	0.375 (0.3130)
ER & ICU	0.030 (0.9410)	0.403 (0.3969)	0.004 (0.9932)	0.143 (0.7213)	−1.479 (0.3976)	0.410 (0.2980)
NCISW	0.090 (0.8219)	0.416 (0.3838)	0.288 (0.5565)	0.257 (0.5233)	−0.464 (0.7643)	0.116 (0.7678)
KM OPD & IPD	−0.101 (0.8080)	0.030 (0.9509)	0.164 (0.7455)	0.024 (0.9547)	0.378 (0.8097)	0.124 (0.7613)
Other	-	-	-	-	-	-
Desire to work in current department						
Yes	−0.033 (0.8049)	−0.119 (0.4517)	0.025 (0.8772)	−0.042 (0.7529)	0.234 (0.6795)	−0.035 (0.7917)
No	-	-	-	-	-	-
Satisfaction with working in current department						
Yes	−0.301 (0.0377)	−0.498 (0.0042)	−0.348 (0.0484)	−0.381 (0.0091)	0.080 (0.8942)	−0.257 (0.0704)
No	-	-	-	-	-	-
Turnover experience						
Yes	0.089 (0.5789)	0.148 (0.4387)	−0.182 (0.3560)	0.024 (0.8811)	0.928 (0.2249)	−0.145 (0.3608)

No	-	-	-	-	-	-
Department transfer experience						
Yes	−0.040 (0.8000)	0.034 (0.8533)	0.132 (0.4921)	0.037 (0.8127)	−0.211 (0.7581)	0.011 (0.9407)
No	-	-	-	-	-	-
Self-rated health						
Bad or below	0.966 (<0.0001)	1.070 (<0.0001)	0.585 (0.0103)	0.878 (<0.0001)	0.693 (0.3746)	0.156 (0.3894)
Normal	0.487 (0.0020)	0.529 (0.0046)	0.455 (0.0171)	0.490 (0.0020)	−0.338 (0.6409)	0.167 (0.2714)
Good or above	-	-	-	-	-	-
Job-focused emotional labor	0.138 (0.3059)	0.063 (0.6930)	0.102 (0.5337)	0.103 (0.4448)	1.103 (0.0788)	0.277 (0.0378 *)

Abbreviations. AN, assistant nurse; ER, emergency room; ICU, intensive care unit; IPD, inpatient department; KM, Korean medicine; NCISW, nursing care integrated service ward; OPD, outpatient department; WM, Western medicine. * $p < 0.05$.

Table S6. Influence of burnout on turnover and medical error (*N* = 117).

	**Medical Error Parameter Estimates (*p*-Value)**				**Turnover Intention Parameter Estimates (*p*-Value)**			
F-value					**2.32**	2.29	2.23	2.30
p-value					0.0059	0.0066	0.0084	0.0066
R^2					0.271	0.268	0.263	0.269
Adj R^2					0.154	0.151	0.145	0.152
Intercept	−4.802 (0.0529)	−4.498 (0.0519)	−3.547 (0.1129)	−4.483 (0.0599)	3.342 (<0.0001)	3.470 (<0.0001)	3.571 (<0.0001)	3.421 (<0.0001)
Clinical experience (years)								
≤6	0.416 (0.6997)	0.456 (0.6727)	0.422 (0.6929)	0.410 (0.7040)	-0.152 (0.5533)	−0.145 (0.5721)	−0.153 (0.5523)	−0.152 (0.5536)
>6	-	-	-	-	-	-	-	-
Education level								
Bachelor degree or above	0.217 (0.7732)	0.165 (0.8279)	0.276 (0.7131)	0.184 (0.8085)	0.058 (0.7380)	0.062 (0.7217)	0.071 (0.6887)	0.057 (0.7475)
Junior college or below	-	-	-	-	-	-	-	-
Income (millions of KRW/month)								
≤2.5	−0.121 (0.8453)	−0.186 (0.7658)	−0.088 (0.8855)	−0.132 (0.8314)	0.189 (0.1732)	0.184 (0.1843)	0.189 (0.1754)	0.186 (0.1804)
>2.5	-	-	-	-	-	-	-	-
Job position								
Assistant nurse	1.320 (0.2807)	1.307 (0.2832)	1.301 (0.2892)	1.333 (0.2767)	−0.336 (0.2155)	−0.341 (0.2097)	−0.341 (0.2108)	−0.336 (0.2155)
Staff nurse	2.075 (0.0929)	2.035 (0.0997)	1.995 (0.1057)	2.009 (0.1044)	−0.122 (0.6617)	−0.133 (0.6335)	-0.134 (0.6335)	−0.134 (0.6302)
Charge nurse or above	-	-	-	-	-	-	-	-
Department								
WM OPD & IPD	0.011 (0.9936)	−0.257 (0.8534)	−0.011 (0.9938)	−0.113 (0.9351)	0.353 (0.3493)	0.321 (0.3974)	0.347 (0.3599)	0.334 (0.3777)
ER & ICU	−2.104 (0.2254)	-2.369 (0.1734)	−2.030 (0.2417)	−2.137 (0.2153)	0.297 (0.4526)	0.261 (0.5109)	0.295 (0.4572)	0.283 (0.4745)
NCISW	−0.725 (0.6397)	−1.008 (0.5201)	−0.698 (0.6525)	−0.851 (0.5841)	0.056 (0.8880)	0.029 (0.9424)	0.051 (0.8990)	0.039 (0.9220)
KM OPD & IPD	0.038 (0.9804)	−0.111 (0.9426)	−0.114 (0.9417)	−0.086 (0.9556)	0.028 (0.9449)	0.009 (0.9818)	0.004 (0.9925)	0.012 (0.9773)
Other	-	-	-	-	-	-	-	-
Desire to work in current department								
Yes	0.228 (0.6876)	0.275 (0.6310)	0.183 (0.7453)	0.225 (0.6917)	−0.066 (0.618)	−0.061 (0.6460)	-0.072 (0.5848)	−0.066 (0.6171)
No	-	-	-	-	-	-	-	-
Satisfaction with working in current department								
Yes	0.243 (0.6901)	0.363 (0.5632)	0.134 (0.8258)	0.285 (0.6451)	−0.220 (0.1350)	−0.212 (0.1574)	-0.238 (0.1061)	−0.216 (0.1478)
No	-	-	-	-	-	-	-	-
Turnover experience								
Yes	0.776 (0.2940)	0.763 (0.3048)	0.826 (0.2660)	0.794 (0.2865)	−0.187 (0.2454)	−0.190 (0.2383)	-0.169 (0.2978)	−0.179 (0.2648)
No	-	-	-	-	-	-	-	-

Department transfer experience								
Yes	−0.157 (0.8202)	−0.197 (0.7757)	−0.175 (0.7973)	−0.188 (0.7854)	0.046 (0.7701)	0.039 (0.8022)	0.036 (0.8195)	0.038 (0.8096)
No	-	-	-	-	-	-	-	-
Self-rated health								
Bad and below	0.402 (0.6302)	0.364 (0.6612)	0.733 (0.3518)	0.444 (0.5908)	0.102 (0.6216)	0.126 (0.5344)	0.191 (0.3130)	0.126 (0.5332)
Normal	−0.493 (0.5009)	−0.509 (0.4858)	−0.373 (0.6035)	−0.498 (0.4952)	0.131 (0.4179)	0.144 (0.3709)	0.168 (0.2929)	0.138 (0.3936)
Good and above	-	-	-	-	-	-	-	-
Personal burnout	0.57 (0.1768)				0.118 (0.2369)			
Work-related burnout		0.568 (0.1280)				0.086 (0.3072)		
Client-related burnout			0.208 (0.5482)				0.048 (0.5618)	
Burnout				0.546 (0.1971)				0.104 (0.2992)

Abbreviations. AN, assistant nurse; ER, emergency room; ICU, intensive care unit; IPD, inpatient department; KM, Korean medicine; NCISW, nursing care integrated service ward; OPD, outpatient department; WM, Western medicine. * $p < 0.05$.
